# Specialized Therapeutic Assessment-Based Recovery-Focused Treatment for Young People With Self-Harm: Pilot Study

**DOI:** 10.3389/fpsyt.2019.00895

**Published:** 2019-12-06

**Authors:** Oliver English, Christy Wellings, Partha Banerjea, Dennis Ougrin

**Affiliations:** ^1^The Institute of Psychiatry, Psychology, Child & Adolescent Psychiatry Department, King’s College London, London, United Kingdom; ^2^Southwark Child & Adolescent Mental Health Service, South London & Maudsley NHS Foundation Trust, Maudsley Hospital, London, United Kingdom

**Keywords:** self-harm, adolescents, Cognitive Behavior Therapy, Solution Focused Brief Therapy, Mentalization Based Treatment, Therapeutic Assessment, Child and Adolescent Mental Health Services

## Abstract

**Background:** Suicide is the second leading cause of death in young people worldwide. Self-harm is the strongest predictor of death by suicide. There is increasing evidence that psychological therapies are efficacious in treating self-harm in adolescents. However, studies so far have predominantly focused on highly selective groups of adolescents and have investigated interventions that require intensive training and considerable expense.

**Methods:** We conducted a pilot study of a novel psychological therapy package, Specialized Therapeutic Assessment-Based Recovery-Focused Treatment (START) that consists of Therapeutic Assessment followed by treatment in one of three modules, depending on adolescents’ needs and preferences: Solution Focused Brief Therapy, Cognitive Behavior Therapy (CBT), or Mentalization Based Treatment. Adolescents (12–17) with at least one self-harm episode in the previous 6 months referred for community treatment, who had no intellectual disability, psychosis or autism were eligible for START. The primary outcome measure was the number of self-harm (regardless of suicidal intent) episodes 6 months before and 6 months after commencing START. Secondary outcomes included measures of psychopathology, functional impairment and family satisfaction.

**Results:** Twenty-one consecutively referred adolescents were recruited and 15 received a therapeutic module of START: three received Solution Focused Brief Therapy, nine CBT, and three Mentalization Based Treatment. There was a statistically significant reduction in the number of self-harm episodes from a mean of 7.93 (SD = 12.26) to 1.00 (SD = 1.47), p < 0.02 following START. There was also a significant reduction in self-harm episodes, Revised Children’s Anxiety and Depression Scale scores and a statistically significant improvement in Children Global Assessment Scale scores for the CBT group alone. There were no significant differences in any other outcomes. Most families were somewhat or very much satisfied with the intervention.

**Conclusion:** The results show that START was associated with a reduction in self-harm and depression and anxiety symptoms, which could indicate that START should be rigorously studied in a randomized control trial (RCT). However, the model had difficulties in its implementation, with CBT being only module that was offered to enough young people to allow before and after analysis. CBT appears to be the most promising module in treating adolescents with self-harm referred to community mental health services.

## Introduction

Self-harm is a significant concern for young people, their carers, and the clinical staff in both physical and mental healthcare services. Studies indicate a prevalence rate of 13.2% for self-harm in 12–18-year olds, and suicide attempt prevalence of 9.7% (1). Self-harm is the strongest predictor of suicide in adolescents (2), and is more prevalent amongst female adolescents than males (3). There have been a substantial debate on how to define self-harm, with US based clinicians and researchers tending to research attempted suicide and non-suicidal self-injury separately (4). However, European based clinicians and researchers often define self-harm as both self-injury and self-poisoning irrespective of suicidal intent (2). UK health services follow the guidelines set out by the National Institute for Health and Care Excellence, who define self-harm in young people over the age of 8 as acts of self-injury or self-poisoning, regardless of their motivations (5).

Despite this substantial concern, research into adolescent treatment is under-investigated, especially following an acute presentation. Over many years, research has suggested that adolescents who engaged in self-harm were less likely to attend further follow-up sessions (6, 7), which has shown to lead to poorer outcomes (8). Adapted from the Cognitive Analytic Therapy model (9), Therapeutic Assessment (TA) is a brief intervention designed to increase treatment engagement of adolescents with self-harm (10). This 30-min intervention after presenting with self-harm led to a significantly improved rate of engagement when compared to assessment as usual, at the 3 month and 2-year follow-up periods (11). However, the inclusion of TA did not lead to a significant difference in psychopathology and functioning scores at 3 months, nor was there a difference in the frequency of accident and emergency department (A&E) self-harm presentations at 2 years (11, 12). Linking TA with interventions likely to reduce self-harm is therefore required.

Recent systematic reviews have highlighted the lack of replicated randomized control trials (RCTs) researching treatment interventions for adolescent self-harm (13–15). These reviews did highlight three interventions in Mentalization-Based Treatment for Adolescents (MBT-A), Cognitive Behavior Therapy (CBT), and Dialectical Behavior Therapy for Adolescents (DBT-A), that significantly reduced the number of self-harm episodes in comparison to the control treatment groups (16–18). More recently, two further RCTs replicated the efficacy CBT/DBT-based family intervention (19) and DBT (20). Additionally, these interventions tended to be delivered in acute services, working with children who have more complex mental disorders than the general population who self-harm (19).

Despite recent improvements in our understanding of the optimal treatment settings (21, 22), supervision (23, 24), and detection (11), there is no evidence that any given intervention is likely to benefit all young people with self-harm. Moreover, young people with self-harm and borderline personality disorder may be more likely to respond to more intensive interventions, such as DBT and MBT (16, 17, whereas young people with substance misuse, anxiety and depression may be more likely to respond to CBT (18). Finally, some young people with self-harm do not meet the diagnostic criteria for any psychiatric disorder and may not require psychological therapies developed to treat psychiatric disorders.

The Treatment of Adolescent Suicide Attempters study (25) focused on predictors of suicidal events during an open treatment trial, having three potential arms of treatment (specialized psychotherapy, medication, or a combination of the two). Although predictors were found and randomization was initially proposed, the open choice format caused the treatment arms to become uneven, with 75% of young people ending up in the combination treatment arm. Treatment choice guided by the young people and their families on one hand and the assessment of the clinical team on the other seems to be an important element in any pragmatic study.

Child and Adolescent Mental Health Services (CAMHS) in the United Kingdom are split into a four-tiered system. Tier 1 include non-mental health specialists, such as general practitioners, teachers, and social. Tier 2 services have mental health professionals within a uni-disciplinary primary care or community services that can treat some mental health disorders and identify more complex mental health needs. Tier 3 services are community multi-disciplinary teams that can treat most complex disorders. Tier 3 services normally capture the widest range of self-harm, from one or two episodes to daily episodes of self-harm. Finally, Tier 4 services are specialist teams, both inpatient and outpatient working with children and young people with the most serious and complex mental health needs.

This article reports the findings for the pilot phase of the Specialized Therapeutic Assessment-Based Recovery-Focused Treatment (START) study, introducing a novel three modular intervention model, aimed to reduce the prevalence of self-harm episodes for adolescents referred to a Tier 3 (standard community multi-disciplinary team) CAMHS in an ethnically diverse inner-city borough of Southwark in London.

## Materials and Methods

### Participants

Participants were all adolescents (12–18 years old) referred to Southwark CAMHS and South London and Maudsley’s (SLaM) Supported Discharge Service with at least one episode of self-harm in the past 6 months between December 2016 and July 2017). The exclusion criteria were: a known intellectual disability (IQ less than 70); immediate need for an inpatient psychiatric admission; a known diagnosis of autism spectrum disorder or psychosis.

### Treatment Interventions and Model

**Figure 1:**
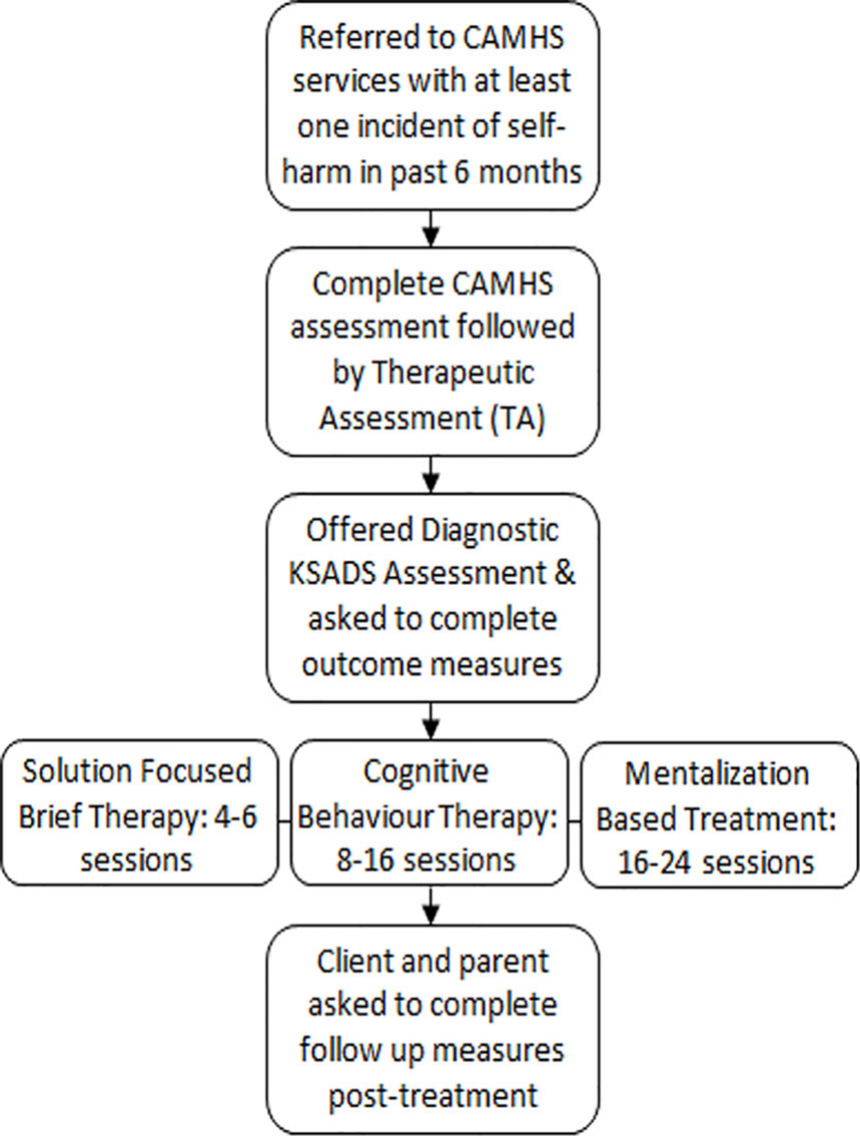
Flowchart of the intervention period.

The START model has been developed in response to the increasing rates of self-harm amongst the adolescent population (26), but also the varying levels of self-harm amongst adolescents and the understanding that someone who has self-harmed once or twice needs a different level of care to someone who is self-harming on a regular basis and alongside other risk taking behaviors. We therefore split the START model into four distinctive interventions (**Figure 1**):

- TA—Once a potentially suitable individual has been identified), the young person completed a full CAMHS assessment followed by the 30-min TA. TA is a collaboratively designed diagram, showing the links between the young person’s reciprocal roles, thought process and “core pain,” their self-harm, and following consequences, feeding cyclically into the young person’s core pain. (10). Through this process the young person discussed their motivation to change, and then looked for and discussed their most favoured way of breaking the created self-harm cycle. After a summation, a therapeutic letter was written based on what was discussed, with the intention to motivate the young person continue to engage with therapy.
- Solution Focused Brief Therapy (SFBT)—For the young person who had no axis I diagnosis or presented as low risk, SFBT was typically chosen. Usually delivered over the course of 4-6 sessions, SFBT focused on the resources the young person already had to help themselves, exploring how they would like their life to be, and what they are doing or can do to work towards this “preferred future” (27).
- Cognitive Behavior Therapy (CBT)—For the young people who self-harm with medium severity, regularity and had a least one axis I diagnosis (anxiety, depression or substance misuse), CBT was offered. The study used the self-harm specific CBT workbook “Cutting Down: A CBT workbook for treating young people who self-harm” (28), the young person was given skills to reduce and eventually stop self-harm over the course of 8-16 sessions.
- MBT-A—For the young person who met or a was close to the diagnostic criteria for emotionally unstable personality disorder (EUPD) diagnosis and/or self-harm with high lethality and frequency (normally daily-weekly self-harm). MBT-A uses the same techniques used in the successful RCT (16), in a treatment ranging from 16 to 24 sessions. For the EUPD population, we chose MBT over DBT (CBT based treatment for EUPD), because DBT requires additional weekly group sessions and phone support that we could not provide in a Tier 3 service. However, many principles and skills in DBT are included in the CBT workbook that was used.

The START model was implemented within a Tier 3 CAMHS community team, as these services receive referrals for all young people with self-harm that cannot be managed by primary care. It would also give a chance to see if the model could be used practically in a real-life setting.

Based on the initial presentation, TA formulation, diagnostic assessment, and their clinical judgement on level of risk, the clinical team at CAMHS and researcher team came together to decide which of the three modules of treatment, if any, was suitable for the young person to move forward with. The young person and their families’ preferences and therapeutic history were considered. During therapy, if the decided module of therapy was proving unsuccessful, a module based on a different therapeutic approach was instead chosen. Initial 2-day training was given by experts in TA and each of the three therapeutic modules (half a day per topic), followed by monthly supervisions for TA and each of the three modules of therapy. All therapeutic models of assessment and therapy were manualized.

### Treatment Objectives

The pilot study is required in order to fully develop the model into a working therapeutic protocol, with the idea that this will form a foundation with which to further investigate the START model into an RCT, providing data for an effect size estimate. Ultimately, the overall objective is to develop START into an evidence-based strategy, and to reduce the prevalence of self-harm within the adolescent population in a Tier 3 community CAMHS setting.

The primary objective is:

- To investigate if the number and severity of self-harm episodes will reduce in the last month of the young person’s time in the Tier 3 community CAMHS.

The secondary objectives are:

- To investigate if the number and duration of inpatient stay will reduce in the 6 months post initial presentation.
- To investigate if overall functioning and psychopathology of the participants will improve following therapeutic intervention.
- To investigate patient and carer satisfaction post therapeutic intervention.

### Therapists

All therapists (N = 14) involved in the study were volunteers who already worked in the multidisciplinary CAMHS team. The experience and background of the therapists varied, with backgrounds in psychiatry, psychology, mental health nursing, and social work. Once recruitment had begun, therapists received monthly supervision sessions in TA and the therapeutic model they were delivering. Some therapists had to attend multiple supervisions if they were administering more than one intervention. Supervisions were 90 min long and were delivered by people qualified to deliver supervisions in their respective models.

### Ethics Approval and Consent to Participate

The study was approved by SLaM clinical audit and service evaluation committee. Consent was given by all adolescents 16 years or over, with consent given along with the adolescent’s assent by the adolescent’s carer.

### Data Collection

Initial assessments were done using the Kiddie Schedule for Schizophrenia and Affective Disorders (KSADS) Present and Lifetime Versions; if a KSADS-Present and Lifetime Versions was unattainable, clinical diagnoses were found using the services electronic medical records system (Electronic Patient Journey System) at SLaM.

Primary outcome measure was the total number of self-harm episodes in the 6-months before and 6 months after the commencement of START. Self-Harm Questionnaire (11) was used to gather information about self-harm episodes pre and post treatment. Any other reported or recorded episodes of self-harm for both the 6 months prior to treatment and 6 months post the beginning of the START package were systematically gathered from the young people, their families the CAMHS electronic medical records system.

Additional outcome measures included the Clinical Global Assessment Scale (CGAS); a clinician rated scale of the young person’s overall functioning, Clinical Global Impressions; a clinician rated scale of the severity of illness that the young person is exhibiting, Strength and Difficulties Questionnaire (SDQ) for both adolescent and carer; a self-report questionnaire assessing strengths and a range of common psychiatric symptoms of the young person and asking if things had improved over the course of treatment, Maclean Screening tool for adolescent and carer; a 10 question screening for EUPD, the Columbia Impairment Scale for adolescent and carer; a self-report questionnaire assessing if the young person has problems at home, school, or socially, Revised Children’s Anxiety and Depression Scale (RCADS) for both adolescent and carer; a 47 question self-report assessment of the young person’s symptoms of anxiety and depression, Child and Adolescent Substance Use Scale; a survey of young people’s and their family’s use of health services over the previous 6 months, and the Health Today segment of the EuroQol-Five Dimensions—Three Levels; a rating from 1 to 100 on the young people’s current health state. These outcome measures were given pre and post intervention, with the addition of the Child and Adolescent Service Experience (ChASE) at follow-up; a questionnaire given at the end of therapy to be completed alone and given back to the research team. All young people and carers were told beforehand that these questionnaires were anonymous and would not be shared with their clinicians. Appointments were logged also using the medical record system, and weekly self-harm rates were also logged by therapists there.

### Intention-To-Treat Analysis

All 21 participants were analysed on the primary and secondary outcomes when possible. Eleven were followed up in person on average 22.1 weeks after their initial assessment, but some form of follow up measure was collected for 20 of the 21 participants. All assessments were done by a researcher who was not blind to the treatment allocations or to the hypotheses of the study.

### Statistical Analysis

To test the distribution of the data, the Shapiro–Wilk test was used. For the normally distributed data, differences between baseline and follow-up measures were analysed using paired sample t-tests. However, if the distribution was nonparametric, paired Wilcoxon signed-rank test was used. Significance was set at p < .05, and all analyses were carried out on SPSS 23.0 (IBM Corporation 2014; Armonk, NY, USA). Individual treatment arms were analysed in the same way where possible.

## Results

### Group Characteristics

Twenty-one young people with self-harm were referred to the service during the pilot period. The demographic characteristics of the 21 young people included in this study are described in **Table 1**, along with the clinical variables. All young people assessed had at least one axis 1 diagnosis. Three young people were on regular psychotropic medication at baseline, with all three on an anti-depressant. At baseline, 11 of the young people had a history of at least one A&E presentation at a hospital in the 6 months prior to coming to the service, 4 had been admitted onto an inpatient unit, and five had been to at least one outpatient CAMHS appointment.

**Table 1:** Patient characteristics (N = 21).

Age range in years (mean)	12.3-17.7 (15.7)
Gender distribution (%)	>17 female, 4 male (81%, 19%)
Ethnicity	>13 white British (61.9%)
	>5 black British (23.8%)
	>1 mixed white/Asian
	>1 white other, 1 other (4.8%)
Diagnosis (%)	
Mood disorders	>12 (57.1%)
Anxiety disorders	>11 (52.4%)
Eating disorders	>4 (19%)
Post-traumatic stress disorder	>2 (9.5%)
Disruptive behavior disorders	>4 (19%)
Other	>1 (4.8%)
Emerging borderline personality	>1 (4.8%)
No. with Axis 1 comorbidity	>10 (47.6%)
Referred from (%)	
Inpatient adolescent unit	>2 (9.5%)
Child and family team	>1 (4.8%)
A&E—7 day follow up	>10 (47.6%)
General practitioner	>6 (28.6%)
Emergency and Pediatric	2 (9.5%)

**Table 2:** Summary of baseline measures.

Outcome measure (n)	Range	Mean (SD)
Clinician measures Children’s Global Assessment Scale (21)	39–72	53.05 (11.25)
Clinical Global Impression—Severity (15)	3–5	4.27 (.70)
Parental Measures Revised Children’s Anxiety & Depression Scale (20)	7–90	44.10 (26.12)
McLean Screening Instrument (14)	1–7	3.86 (1.88)
Columbia Impairment Scale (14)	6–42	23.64 (10.55)
Strengths & Difficulties Questionnaire (20)	8–24	15.55 (5.07)
Client Measures Revised Children’s Anxiety & Depression Scale (21)	34–120	70.24 (25.26)
McLean Screening Instrument (14)	2–10	6.93 (2.46)
Columbia Impairment Scale (14)	12–36	26.50 (7.39)
Strengths & Difficulties Questionnaire (20)	12–27	20.25 (4.40)
Health Today (14)	10–75	44.50 (17.25)
Self-harm episodes (15)	0.2–30	9.40 (13.12)

### Service Use

**Figure 2:**
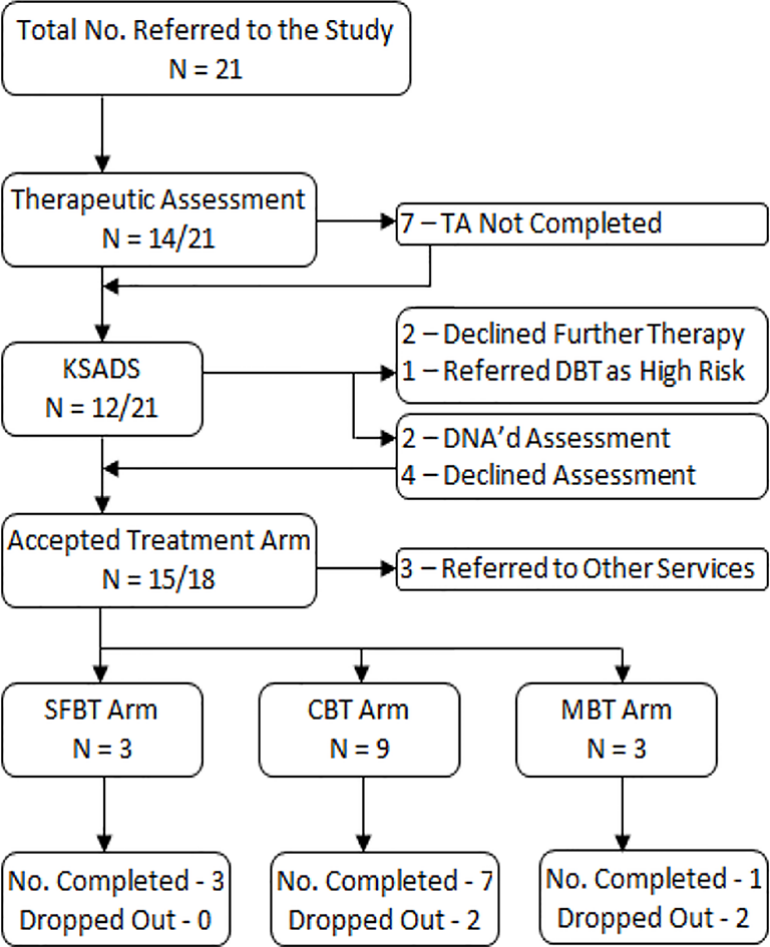
Progression of clients through the intervention.

As shown in **Figure 2**, 15 young people started one of the three treatment arms, the majority were offered CBT. However, every adolescent had some form of intervention, with eight young people going through the intervention in its entirety. Three young people completed the TA before dropping out, with two declining further therapy afterwards and one self-harming severely immediately before the following session; with the latter the team decided to refer them to the Tier 4 DBT service. Two young people completed just the diagnostic KSADS and one adolescent attended both a TA and KSADS appointment, but from the diagnoses given using the KSADS the adolescent team decided that all three would be better treated away from the model (one was referred to an obsessive compulsive disorder clinic, one was treated for attention-deficit/hyperactivity disorder which appeared to have been a clinical priority, and another was referred to an eating disorder service). Finally, four young people went straight into treatment, two adolescents went from the TA directly to treatment, and one young person missed the TA but did attend the KSADS appointment and had treatment. Baseline measures for the young people entering the service can be seen in **Table 2**.

**Table 3** shows the mean and range of attendance rates between the three treatment modules. SFBT and CBT groups average attendance rate was within the expected treatment range, with MBT seeing fewer sessions than expected. SFBT had the highest rate of not attending amongst the groups, despite young people expecting to only attend 1–4 sessions.

**Table 3:** Summary of session attendance.

Treatment	Attended sessions		Missed sessions	
	Range	Mean (SD)	Range	Mean (SD)
SFBT	2–7	4.33 (2.52)	1–4	2.33 (1.53)
CBT	1–24	10.67 (7.48)	0–5	1.89 (1.83)
MBT	2–11	6.33 (4.51)	0–1	0.33 (.58)
Total	1–24	8.53 (6.60)	0–5	1.67 (1.68)

SFBT, Solution Focused Brief Therapy; CBT, Cognitive Behavior Therapy; MBT, Mentalization-Based Treatment.

Where three of the young people were on anti-depressant medication when they entered into the study, at follow up a new young person was now on an anti-depressant, along with two of the original young people; the other adolescent moved from an anti-depressant to an anti-psychotic. We received follow-up data for 14 of the 16 adolescents we initially received baseline data on. Inpatient admissions reduced from four at the 6 months prior to baseline assessment to two in the 6-month following the commencement of treatment, with one young person having an admission pre and during intervention. Finally, A&E 6-monthly presentations to a hospital reduced from 11 young people to four, with all four young people having had an A&E presentation at baseline as well. Additionally, three of the clients with an A&E presentation were for self-harm or suicidality, with the fourth for alcohol poisoning.

### Clinical Outcomes

**Table 4** summarizes the group measures at the end of the modular intervention period (16.3 weeks on average). **Table 5** shows the changes in score from baseline till the end of intervention, with the last month score (primary outcome), and the adolescent RCADS showing significance changes in scores (P < .05). We did not have enough data for looking at the effectiveness of SFBT or MBT alone, but **Table 6** shows the changes in score from baseline till the end of the intervention period for the young people given CBT. Here, monthly self-harm average was shown to have a significant change in scores, with significant changes in scores (P < .05) also seen again with the adolescent RCADS and with the therapist rated CGAS.

**Table 4:** Clinical Measures at the end of intervention.

Outcome measure (n)	Range	Mean (SD)
Clinician measures Children’s Global Assessment Scale (20)	41–80	60.50 (12.56)
Clinical Global Impression—Severity (13)	2–5	3.46 (.88)
Parental Measures	2–77	32.80 (22.27)
Revised Children’s Anxiety & Depression Scale (15)		
McLean Screening Instrument (11)	0–8	3.82 (3.13)
Columbia Impairment Scale (11)	5–40	21.45 (12.24)
Strengths & Difficulties Questionnaire (13)	6–21	14.38 (5.61)
Client Measures	7–90	47.92 (26.03)
Revised Children’s Anxiety & Depression Scale (13)		
McLean Screening Instrument (11)	0–9	5.09 (2.47)
Columbia Impairment Scale (11)	9–40	23.18 (8.76)
Strengths & Difficulties Questionnaire (13)	4–28	17.62 (6.56)
Health Today (11)	30–90	56.91 (22.79)
Self-harm episodes (14)	0–5	1.01 (1.57)

**Table 5:** Paired means and significance of outcomes.

Outcome measure (n)	Baseline Mean (SD)	Follow-up Mean (SD)	Sig
Clinician measures	53.45 (11.38)	60.50 (12.56)	.056
Children’s Global Assessment Scale (20)			
Parental Measures	39.33 (26.49)	32.80 (22.27)	.257
Revised Children’s Anxiety & Depression Scale (15)			
McLean Screening Instrument (11)	4.00 (2.00)	3.82 (3.13)	.819
Columbia Impairment Scale (11)	24.27 (10.81)	21.45 (12.24)	.420
Strengths & Difficulties	15.62 (5.41)	14.38 (5.61)	.456
Questionnaire (13)			
Client Measures			
Revised Children’s Anxiety & Depression Scale (13)	75.31 (26.80)	47.92 (26.03)	.006*
McLean Screening Instrument (11)	6.73 (2.61)	5.09 (2.47)	.158
Columbia Impairment Scale (11)	26.64 (7.00)	23.18 (8.76)	.331
Strengths & Difficulties Questionnaire (13)	20.62 (4.66)	17.62 (6.56)	.050
EQ5D Health Today (11)	48.36 (16.97)	56.91 (22.79)	.244
Self-harm episodes (14)	9.26 (12.05)	1.01 (1.57)	.018*

Fifteen participants gave a Clinical Global Impressions score at the beginning of treatment, with majority of therapists giving a score of either moderately or markedly ill (13 of 15). At follow up, almost all therapists registered some form of improvement in their young people (12 of 13).

**Table 6:** Paired means and significance of outcomes for Cognitive Behavior Therapy alone.

Outcome measure (n)	Baseline Mean (SD)	Follow-up Mean (SD)	Sig
Clinician measures Children’s Global Assessment Scale (20)	50.00 (9.33)	57.22 (11.10)	.046*
Parental Measures Revised Children’s Anxiety & Depression Scale (15)	49.78 (24.96)	46.33 (18.07)	.664
McLean Screening Instrument (11)	3.63 (2.00)	4.00 (3.02)	.685
Columbia Impairment Scale (11)	23.00 (7.48)	22.50 (11.48)	.735
Strengths & Difficulties Questionnaire (13) Client Measures	15.75 (5.60)	15.00 (5.10)	.761
Revised Children’s Anxiety & Depression Scale (13)	79.78 (24.09)	49.11 (29.55)	.027*
McLean Screening Instrument (11)	6.13 (2.80)	4.63 (2.72)	.336
Columbia Impairment Scale (11)	24.25 (6.61)	21.88 (9.78)	.624
Strengths & Difficulties Questionnaire (13)	19.88 (4.52)	15.88 (6.53)	.071
EQ5D Health Today (11)	55.50 (10.85)	57.75 (22.02)	.758
Self-harm episodes (14)	14.06 (14.04)	1.06 (1.63)	.027*

*P > .05.

### Patient and Carer Satisfaction

At the end of the intervention period, patient satisfaction was rated using two questions in the follow up version of the SDQ, and the ChASE. In the SDQ, most carers (9 of 12) and adolescents (8 of 12) who gave feedback responded that they or their child were a bit or much better post intervention, with no one stating that they had become worse. All carers and adolescents felt that the service had been helpful in other ways.

11 adolescents completed the ChASE questionnaire at the end of intervention, and most of the adolescents (8 of 11) found that the appointments helped them get on with their life most or all of the time. Additionally, all of the adolescents who responded felt that they could trust their therapists (eight felt that this was all of the time), felt that their therapist really understood them (six said all of the time), and felt that their therapist was kind and caring (10 said all of the time).

## Discussion

The pilot study’s primary objective was to see whether this treatment model could successfully reduce the number and severity of self-harm in adolescents presenting to a Tier 3 CAMHS team. For the total project, it was found that the monthly self-harm average reduced significantly following intervention. There was also a statistically significant reduction in the monthly self-harm average post intervention for CBT module alone. Regarding the severity, 68.8% of the young people recorded had been to the A&E of a hospital in the 6 months prior to treatment, all of which were for self-injury or self-poisoning. Post intervention this reduced to 28.6%, 21.4% for the young people attending for self-injury and self-poisoning incidences. This is line with the findings by 29.

Our secondary objectives were to investigate inpatient admissions, and overall functioning, psychopathology and patient satisfaction. The number of inpatient admissions for this pilot study was small, partly from the small sample size and response rates, but we did see inpatient admissions reduce from 25% of recorded young people, to 14.3%.

The total score for RCADS showed that the total anxiety and depression score significantly reduced post intervention for both the combined interventions and CBT module alone condition. The reduction of this score is even more significant when considering that 17 of the 21 participants were given either an anxiety or depression related diagnosis. Another measure with significant pre-post change was CGAS for the CBT module alone condition. All other measures showed no statistically significant pre-post differences. Adolescents consistently scored themselves as more impaired than their carers scored them, with the adolescents also seeing a greater change in the scores at the end of the intervention period. This was also seen in Ougrin etal. (11) in a similar population.

Finally, the study found that patient and carer satisfaction was overall positive, with most adolescents and their carers feeling that the intervention was somewhat or very much helpful.

Several limitations apply to this study. Small sample size, high dropout rate and treatment allocation led by clinical team are key. The therapists, the young people, their family members and the researcher were not blind to the hypothesis of this study or the treatment module allocations. This could be a challenge when moving into an RCT phase of research, as the allocation of young people to a specific module, SFBT, CBT, or MBT required input from the clinical team. Standardising module allocation might address this problem.

We did not anticipate that only three young people would be allocated to the MBT and SFBT arms of the study. For MBT, this may be partly explained by the availability of a Tier 4 DBT service within the trust that meant that most young people who could have been allocated to MBT were referred to DBT directly. An RCT being implemented at multiple sites across the country, most of which have no DBT service might address this limitation. For SFBT, again with an area that doesn’t have the resources of South London and Maudsley NHS trust, more young people could have been referred to a Tier 3, community and multidisciplinary service). The sample size was also smaller than anticipated, however, the study was pragmatic and undertaken in a real-life community setting, which with typically high level of drop outs or onward referrals to more specialist services.

The study was not a RCT and we cannot exclude the passage of time as a factor in the reduction of self-harm pre-and post-intervention (30). The decision to allocate the young person to one of the three treatment modules was not fully standardized and the multidisciplinary team had the final decision-making power on which module to offer to the individual young person, considering the wishes of the family and the results of the TA. As highlighted in Brent and colleagues (25), taking the wishes of the young person and family can skew the amount of young people allocated to each treatment arm. Whereas this introduces a potential bias, this procedure closely follows real-life treatment allocations in standard community services. A problem with implementing this model in a real-life community service setting is that there may be an unpredictable changes such as staff turnover, and during the recruitment phase there was an unusually high rate of turnover. This certainly slowed recruitment during this period, as new therapists had to be identified and trained. For a future RCT a plan would need to be in place in order to train new staff quickly. However, with all the treatment modules being manualized and having monthly supervisions, new therapists could be trained quickly.

Although TA is an integral part of the START model, this was not individually assessed in the pilot. For a future RCT, TA needs to be evaluated for potential effects on engagement and other outcomes seen in previous studies (11).

For future research, we have several options available for potential RCTs or further pilot studies. As well as completing the START model in full, the judgement of the clinical team favoured the CBT intervention. However, we don’t know if MBT or SFBT would have worked just as well for that group, as we were not making inter-group comparisons. Another option is that START could be adapted into a step-based model, which could be implemented in several ways. One option was that everyone receives TA, followed by SFBT, and it is felt by the clinical team and young person that they required more therapy, they would move onto CBT, and then MBT, if even more/another approach was required. Yet another option is TA, followed by SFBT, followed by MBT or CBT if more therapy is required. This was proposed because SFBT has a significantly shorter treatment length and was anecdotally popular with the clinicians, especially for young people with less complex presenting problems. Any step-based pathway should be revisited in another pilot/feasibility study.

## Conclusion

The results of this pilot study show that START could be successfully implemented in an inner-city ethnically diverse community mental health service and associated with a reduction in self-harm in young people. This model requires thorough investigation in RCTs, following which this approach may become a feasible tool for other multidisciplinary community services in the UK and elsewhere. CBT appeared to be a promising modality in this setting, however, other modalities need to be further investigated in the settings with poorer access to specialist teams and with teams looking after young people with less severe presentations.

## Data Availability Statement

The datasets used and analysed during the current study are available from the corresponding author upon reasonable request.

## Ethics Statement

The study was approved by the SLaM Clinical Audit and Service Evaluation Committee. Consent was given by all adolescents 16 years or over, along with consent by the adolescent’s carer.

## Author Contributions

OE analysed, interpreted the patient data, and was the primary contributor towards the manuscript. DO oversaw the work completed, edited the manuscript as well as contributing towards it. CW and PB read and approved the final manuscript.

## Funding

This study was funded by the Guy’s & St. Thomas Charity, reference EFT150701.

## Conflict of Interest

The authors declare that the research was conducted in the absence of any commercial or financial relationships that could be construed as a potential conflict of interest.
